# The COPEWELL Rubric: A Self-Assessment Toolkit to Strengthen Community Resilience to Disasters

**DOI:** 10.3390/ijerph16132372

**Published:** 2019-07-04

**Authors:** Monica Schoch-Spana, Kimberly Gill, Divya Hosangadi, Cathy Slemp, Robert Burhans, Janet Zeis, Eric G. Carbone, Jonathan Links

**Affiliations:** 1 Johns Hopkins Center for Health Security, Johns Hopkins Bloomberg School of Public Health, Baltimore, MD 21202, USA; 2 Disaster Research Center, University of Delaware, Newark, DE 19716, USA; 3 Independent Consultant; 4 Chester County Department of Emergency Services, West Chester, PA 19380, USA; 5 US Centers for Disease Control, Center for Preparedness and Response, Atlanta, GA 30333, USA; 6 Johns Hopkins University Center for Public Health Preparedness, Johns Hopkins Bloomberg School of Public Health, Baltimore, MD 21205, USA

**Keywords:** community resilience, disaster, measurement, assessment, social capital

## Abstract

Measurement is a community endeavor that can enhance the ability to anticipate, withstand, and recover from a disaster, as well as foster learning and adaptation. This project’s purpose was to develop a self-assessment toolkit—manifesting a bottom-up, participatory approach—that enables people to envision community resilience as a concrete, desirable, and obtainable goal; organize a cross-sector effort to evaluate and enhance factors that influence resilience; and spur adoption of interventions that, in a disaster, would lessen impacts, preserve community functioning, and prompt a more rapid recovery. In 2016–2018, we engaged in a process of literature review, instrument development, stakeholder engagement, and local field-testing, to produce a self-assessment toolkit (or “rubric”) built on the Composite of Post-Event Well-being (COPEWELL) model that predicts post-disaster community functioning and resilience. Co-developing the rubric with community-based users, we generated self-assessment instruments and process guides that localities can more readily absorb and adapt. Applied in three field tests, the Social Capital and Cohesion materials equip users to assess this domain at different geo-scales. Chronicling the rubric’s implementation, this account sheds further light on tensions between community resilience assessment research and practice, and potential reasons why few of the many current measurement systems have been applied.

## 1. Introduction

Reasons to reform society’s relationship to the environment continue to accumulate: a changing, increasingly volatile climate; more frequent disasters and disease outbreaks; and a population growing in number and income inequality [1,2,3,4,5,6]. In this severe context, practitioners, policymakers, and affected communities have converged around the notion of “community resilience,” recognizing that they cannot sustain a business-as-usual outlook and they need to take a more proactive approach to reducing risks [7]. That desire has generated many differently inflected definitions, owing to malleability of the term, community, and the intangibility of resilience [8,9,10,11]. Common to physical, ecological, social and psychological sciences, resilience has most often denoted an ability to regain functionality or “bounce back” after a major stressor [12,13,14]. Other meanings include social learning and adaptation—the ability to anticipate an event and to make post-disaster adjustments to improve communal life and reduce future risk, or “bounce forward” [7,15,16]. 

More recently, however, an abundance of definitions has given way to a surfeit of frameworks, models, and measures [17,18,19,20,21,22,23,24,25,26,27,28,29]. Transforming community resilience aspirations into actions and outcomes has thus become less a challenge of defining the concept than operationalizing it [17,18,19]. Measurement schemes differ widely in what is being measured—a variability due to several factors including the multifaceted nature (e.g., social, natural, built, and economic components) and varying scale (e.g., neighborhood, city, region, nation) of a community unit; the differential treatment of resilience as a process, outcome, or both; concern with a particular hazard, multiple hazards, or both shocks and acute/chronic stressors; and the interests and priorities of different model makers and users [18,20,21].

As several critical reviews suggest, existing methodologies for measuring or assessing community resilience demonstrate a lack of universal metrics [22,23,24]. For instance, some schemes are sector-specific (e.g., energy, housing, transportation); others adopt a multi-sector, whole-of-community approach. Some address a specific hazard threatening a certain place (e.g., earthquake country, tornado alley); still others consider multiple hazards impinging on the community. Focused at a certain scale, some metrics are not generalizable to other community units. Employing an engineer’s vantage, some methodologies zero in on physical structures; others, informed by the social and behavioral sciences, spotlight organizational and interpersonal dimensions. 

Apart from what is being measured, assessment approaches also diverge in terms of how community resilience characteristics are measured, by whom, and for what purpose [17,18,19,20,30,31,32]. “Top-down” methodologies, also termed “objective” approaches, typically involve an externally determined framework, implementation by authorized experts, and reliance on quantitative indicators, all of which allow for controlled comparisons across communities. By contrast, “bottom-up” or “subjective” methodologies enlist community members in developing a context-specific perspective on resilience, integrating experiential knowledge into the evaluation, and generating greater accountability in the local application of the results. Those taking stock of the two approaches argue that in combining standard and tailored measurements, communities can benefit from the strengths of both [20,31,32]. 

Despite their abundance in number and scope, and despite the many advantages they can confer, community resilience measurement methods nonetheless do not exhibit a strong track record of implementation [17,21,22,23,29]. Few community resilience metrics have been applied in more than one community or more than one time in the same community [21]. Although not yet fully enjoyed by many localities, a principal value of resilience assessment is enabling communities to map their resilience, understand gaps, prioritize concerns, identify leverage points for intervention, and instill accountability for taking corrective action [17,18,19,23,26,27,31]. Assessment approaches also serve important communication, persuasion, and social mobilization purposes, as individuals come to see (and enjoin others to see) community resilience as a high-value and doable objective [18,23,25,26,30]. 

Critical appraisals of the limited use of existing indicators suggest that resilience science and practice have overlapping goals, i.e., the transformation of a complex phenomenon into a discernible object, the state of which can be manipulated though human intent and community resources [32]. Nevertheless, pulling researchers and practitioners in separate directions still are their respective professional aims: robustly characterizing community resilience versus materially improving local resilience [21,28,29]. Factors impeding the greater use of existing indicators include a mismatch with local risk realities and decision-making processes, their computational complexity and associated costs (e.g., personnel, time) in managing that, and an absence of follow-on guidance about resilience-enhancing interventions [17,21,22,28,33,34]. To overcome these and other yet-to-be uncovered obstacles, surveyors of the field are calling for more empirical studies of the actual conduct of community resilience assessment and for earlier/closer collaborations between indicator developers and users [21,24,26,28,32]. 

**Figure 1:**
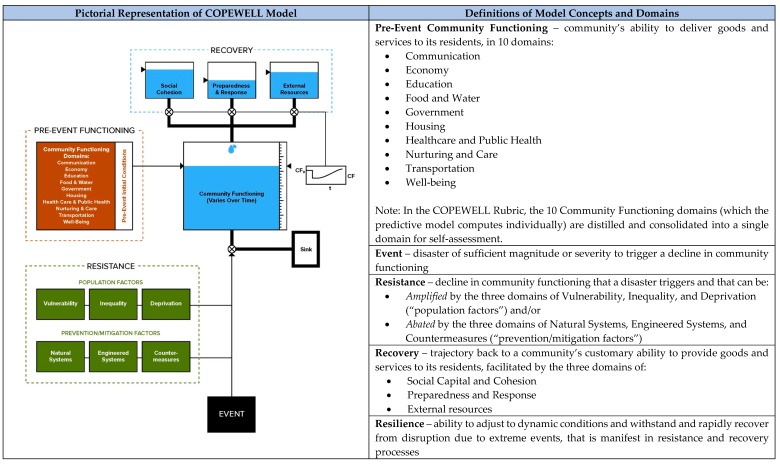
Composite of Post-Event Well-being (COPEWELL), A Conceptual and Computational Model for Predicting Post-Disaster Community Functioning and Resilience–Pictorial and Prose Representations (Adapted from References [35,36]).

In light of the current state of resilience measurement science and practice, this article’s purpose is threefold. The first is to contribute to the growing literature on resilience and measurement by documenting the COPEWELL Rubric, a participatory process of community resilience assessment and action planning that was co-developed with local users and national thought leaders. The COPEWELL Rubric derives from the Composite of Post-Event Well-being (COPEWELL), a conceptual and computational system dynamics model for predicting community functioning and resilience after disasters (Figure 1), developed through research supported by the Centers for Disease Control and Prevention (CDC) [35,36]. The second aim is to close the gap in empirical studies of the application of community resilience assessment tools [24,26]. Prior academic reporting on resilience measurement has tended to privilege substantive (i.e., analytic content) over procedural (i.e., implementation process) matters [28,32], although more detailed case studies of application are emerging [37,38]. This article, thus, relates work with community partners in applying a self-measurement scheme and the learnings about implementation gleaned during this collaboration. The third objective is to uncover traits that communities do or do not desire in a resilience self-assessment toolkit, thus providing further insights into the competing and/or overlapping requirements of community resilience research and practice.

## 2. Materials and Methods

### 2.1. Materials

The original COPEWELL model represents the complex interplay of systems that influence resilience, and it predicts community resistance, recovery, and post-event functioning (i.e., the ability to provide goods and services) following natural and man-made disasters [35,36]. Drawing from the extensive disaster literature, the model identifies 10 domains (e.g., housing, transportation, health care/public health) underpinning the community’s vitality. The model also incorporates domains that either dampen (e.g., natural/engineered systems) or augment (e.g., population inequality/deprivation) the magnitude of effect an event has on a community’s initial loss of functioning (reflecting the community’s “resistance”), and domains (e.g., social cohesion, preparedness and response, external resources) that help replenish or restore community functioning following the event (reflecting the community’s “recovery”). Resistance and recovery jointly manifest resilience. The model’s main output is the predicted post-event time-course of community functioning; its current inputs are publicly available, county-level data in the United States (US). Model outputs are visualized using national maps that allow counties to compare themselves to each other and that enable national decision makers to discern more readily any unevenness in resistance, recovery, and resilience to disasters across the country. 

**Figure 2:**
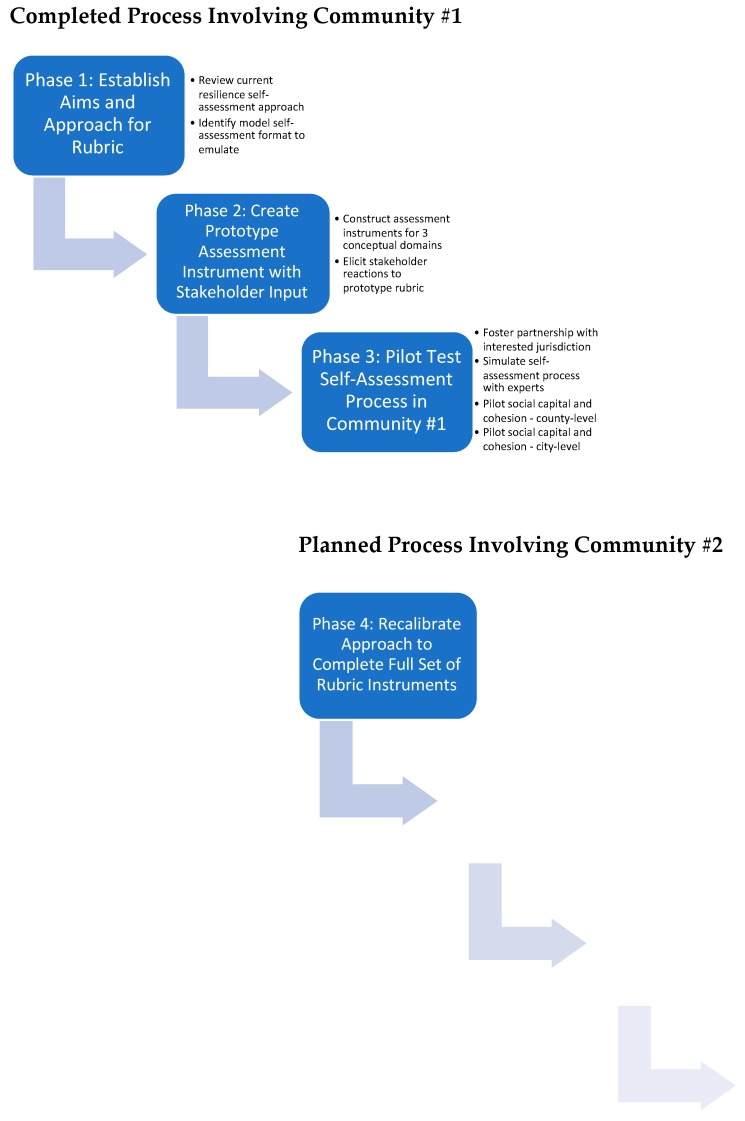
COPEWELL Rubric Co-Development Process.

Whereas the COPEWELL model provides a bird’s eye view of factors affecting community resilience nationwide, the rubric—whose co-development and community implementation are chronicled below (Figure 2)—offers an eye-level perspective on a specific locality’s ability to anticipate, withstand, recover after, and learn from a disaster. The rubric was envisioned as a way to overcome certain limitations of the original COPEWELL model. County-level data, for example, were not available or of sufficient quality to populate all domains of the COPEWELL model in a complete fashion. Moreover, counties constituted only one kind of community, and resilience-related advocates or decisions might not correspond neatly to this geographic unit. Lastly, the externally-generated, comparative snapshots afforded by COPEWELL might not constitute the best or the only incentive that could inspire and help individual communities—with their diverse risks, populations, and histories—to plan and take concrete steps to enhance their resilience. Therefore, the investigators committed to co-develop and test—with community-level users—a self-assessment toolkit that would be flexible regarding geo-scale, use self-identified data sources, and foster greater local ownership of the resilience enterprise.

### 2.2. Development Process

#### 2.2.1. Establish Aims and Approach for Rubric—Phase 1

Review current resilient self-assessment approaches: The COPEWELL team—a 20-person multidisciplinary panel comprised of researchers and subject matter experts from fields that include civil engineering, public health, public policy, emergency management, risk management, systems modeling, and the social and behavioral sciences—tasked a small workgroup to spearhead rubric development. Constituting the 5-person rubric workgroup were 2 social science researchers with expertise in community resilience and community engagement, 2 public health practitioners with prior experience in agency leadership roles and with community engagement for disasters, and 1 researcher on public health policy and preparedness. A first step was to gather information on leading rubric-based, community-led tools for assessing resilience to disasters [39,40,41,42,43]. Review goals were to identify the limitations and strengths of current resources and to consider how a resilience self-assessment whose evaluation domains are informed by the conceptual framework of the COPEWELL model [35] could add a unique contribution to the field. 

**Figure 3:**
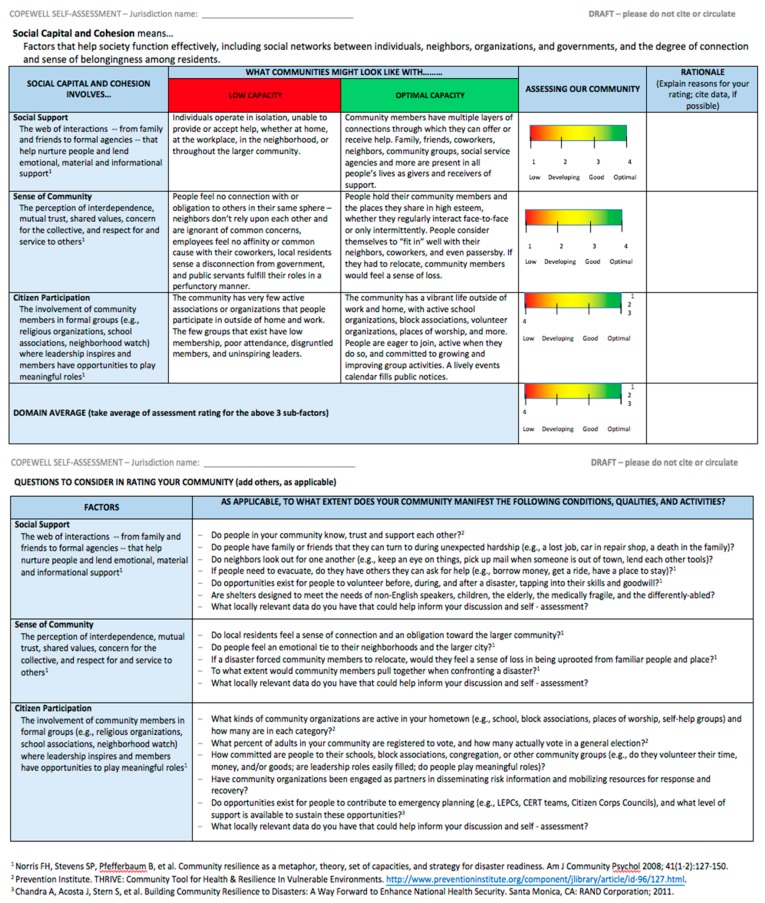
Self-Assessment Instrument for the Social Capital and Cohesion Domain of the COPEWELL Rubric. LEPCs—Local Emergency Planning Committees; CERT—Community Emergency Response Team.

Identify a model self-assessment format to emulate: After reviewing a model self-evaluation tool with the COPEWELL team and discussing structural features to adapt or avoid [44], the workgroup identified the key parts of a COPEWELL self-assessment instrument (see Figure 3). These were: a definition of the conceptual domain being evaluated; a list and definitions of domain “sub-factors”; qualitative descriptions of optimal- and low-capacity levels in the community for that domain; open-ended questions to guide self-assessment and reflection; rating scales; a domain average rating; and (eventually) a list of concrete steps for strengthening each domain (see Figure 3 for concrete illustrations). The workgroup included prompting questions as a way of encouraging stakeholders to identify data to support their assessment. For “rationale,” users were to add context, rating justifications, and other information to enable others to understand the basis for the scores. 

#### 2.2.2. Create Prototype with Stakeholder Input—Phase 2

Construct assessment instruments for 3 conceptual domains: The workgroup selected Healthcare and Public Health (HPH), Natural Systems (NS), and Social Capital and Cohesion (SCC) as the first domains with which to apply the new template. These domains are associated with overarching COPEWELL concepts, namely, community functioning (HPH), resistance (NS), and recovery (SCC) (Figure 1) and draw from distinct literatures. To develop instrument content, the workgroup reviewed pertinent literature, extracting and rephrasing descriptive and prescriptive information for each assessment. The SCC instrument drew, for instance, upon leading research and practice treatments of social capital as a factor in community resilience [11,45,46]. The full workgroup and COPEWELL team then reviewed draft instruments.

Elicit stakeholder reactions to prototype: On 6 March 2017, the COPEWELL team hosted a one-day stakeholder workshop in Baltimore, Maryland to explore the COPEWELL Rubric’s potential. In attendance were experts in emergency management and public health (*N* = 13) from academic, non-profit, and governmental (federal, state, and local) sectors, including those with assignments in community preparedness, community resilience, and evaluation and research. Workshop objectives were to obtain preliminary user reactions to the structure and substance of sample rubric domains; identify real world implementation challenges and facilitators of success; and gauge the type and degree of technical assistance needed locally to implement the rubric. 

#### 2.2.3. Pilot Test the Community Self-Assessment—Phase 3

Foster a partnership with an interested local jurisdiction: Following the 6 March 2017 workshop, COPEWELL project leads and rubric workgroup members spoke with stakeholders interested in piloting the model and rubric locally. These exchanges initiated a series of calls and meetings during which COPEWELL and Chester County, PA (a March 2017 workshop attendee) explored, launched, and cemented a partnership. Located in southeast Pennsylvania in the Philadelphia Metropolitan Area, Chester County includes 759 square miles, 73 municipalities, and roughly 500,000 residents. Well-resourced, the county nonetheless has pockets of economic distress and uneven access. The county’s Emergency Services Department community resilience coordinator has served as the partnership’s local champion, drawing in a variety of governmental and nongovernmental collaborators. Workshops were held 17 November 2017, 23 January 2018, and 13 April 2018 to review Chester County’s ongoing resilience efforts and data sources and elicit ideas about how to create synergy with COPEWELL. Community partners in Chester County identified SCC as the domain within the COPEWELL model that they most wanted to assess, given local interests and resilience-strengthening efforts already underway in the county.

Simulate community resilience self-assessment with experts: In preparation for the Chester County pilot, the workgroup simulated a community-based discussion using the COPEWELL Rubric to rate a jurisdiction’s capacity in a specific domain. This mock self-assessment exercise was part of a 90-minute workshop on the COPEWELL Rubric at the 2018 Annual Public Health Preparedness Summit in Atlanta, GA, convened by the National Association of County and City Health Officials on 17 April 2018. Summit attendees were public health preparedness professionals and included researchers, practitioners, and policymakers from all levels of government. Workshop participants broke into 3 groups of 10 people; an individual rubric workgroup member facilitated each group tasked with assessing one of three domains (SCC, HPH, NS). After the simulated self-assessments, attendees critiqued the specific instruments in terms of basic design and usability, commenting on future field implementation. This session presented the opportunity to prepare an agenda (Figure S1), participant guide (Figure S2), and orientation slides that could be repurposed for future community use.

Pilot social capital and cohesion instrument with a community (county-level): On 27 July 2018, the rubric workgroup, in collaboration with the Chester County community resilience coordinator, convened a 2-hour session at county-owned facilities in West Chester, PA among 17 individuals who represented a range of county and municipality agencies (e.g., planning, public health), nonprofits (e.g., visually impaired, community health), and utilities (e.g., energy, water). Key informants engaged in a facilitated community-based discussion to rate county capacity around SCC, stimulating ideas about how to strengthen the domain and identifying priorities and players for doing that work. Exercise objectives were to gain insights into how the SCC instrument could be adapted for use by local Chester County communities that have varying levels of interest and expertise in resilience, and to elicit practical feedback on process improvements and supporting materials that the COPEWELL project will need to develop so that communities can apply the rubric independently. Pre-prepared session materials included an agenda (Figure S3), moderator’s guide (Figure S4), opening slides (projected), and evaluation form.

Pilot social capital and cohesion instrument at another geo-scale (city-level): On 18 October 2018, the rubric workgroup, in collaboration with the community resilience coordinator, convened another 2-hour community-based SCC assessment session at the senior center in Coatesville, PA—an African-American majority municipality in Chester County, with a population of 13,132 people and a $36,212 median income (2017) compared to the county’s median income of $88,995 (2017). The 18 community attendees were comprised of local community-based organization leaders, in addition to several community members. Based on feedback at the July 2018 self-assessment session, meeting materials were revised (Figures S5 and S6); in addition, paper handouts were used in lieu of a projector. Session objectives were to engage residents in rating, as a group, Coatesville’s ability to pull together as a community; motivate a dialogue about what actions Coatesville can take to strengthen everyday neighborliness and community participation; and enable the COPEWELL team to discover needed process and product improvements.

## 3. Results

The following represent recurrent and/or weighty issues that emerged during the co-development process:

### 3.1. A New Community Resilience Assessment Tool Must Provide Comparative Advantages

Phase 1 review of existing self-assessment tools suggested that the new rubric would add value by providing communities a novel way of thinking about resistance, recovery, and resilience (i.e., the COPEWELL model) and by combining social, natural, and physical elements into a comprehensive picture where other resilience assessment tools have tipped the scales either to social or physical factors [23,24]. The COPEWELL model, upon which the rubric’s framework is based, recognizes community functioning domains that need to remain uninterrupted in a disaster (e.g., communication, housing, food and water, healthcare and public health). The model also integrates domains that either abate (e.g., engineered systems, medical countermeasures) or amplify (e.g., population vulnerability and deprivation) the effect an event has on a community’s initial drop in functioning (representing “resistance”), and it includes domains (e.g., social capital and cohesion, preparedness and response) that help replenish community functioning post-event (representing “recovery”) [35]. Together, resistance and recovery comprise resilience. Phase 2 stakeholders indicated that in a field crowded with assessment tools, the COPEWELL Rubric would need to show a comparative advantage to encourage its use.

### 3.2. Communities, Especially Low-Capacity Ones, Require Some Degree of Technical Assistance

Phase 1 document review revealed that communities using other self-assessment tools often receive external assistance. For example, in the case of the Community Resilience System, pilot communities received help from developers in the form of calls, in-person meetings, and webinars; support for startup also included self-help videos, slide decks, brochures, and other materials [41,42]. At the Phase 2 stakeholder engagement workshop, attendees noted that many of the current tools for community resilience self-assessment “sit on the shelf and gather dust” because local jurisdictions do not have the capacity to implement them. Phase 3 piloting indicated the need for detailed user guides to enable communities’ autonomous self-assessment (e.g., sample agendas, detailed moderator script, process options, slide decks to introduce concepts, list of suggested participants). Divergent A/V capabilities as well as room size and set up—as seen across the three field test sites—in a small way spoke to the range of capacity that different jurisdictions may have to apply the rubric.

### 3.3. Community-Based Self-Assessment Tools Must Strike a Balance between Academic Precision and Broad Intelligibility and Avoid Terms That Have Unhelpful Connotations

During Phase 2, workgroup members developing individual self-assessment instruments drew upon the scientific literature to represent the domain accurately, keeping with current theory and evidence. As a result, the SCC instrument incorporated three sub-factors: social support, sense of community, and citizen participation. During Phase 3 field tests of the SCC instrument, participant reactions and comments suggested the need for changes. Preparedness summit attendees, for instance, advocated using “everyday language” and replacing the phrase “citizen participation” with “community involvement” to be more inclusive. During Chester County discussion of the SCC rubric, the lines blurred between social support and sense of community, with individuals lumping these characteristics together. The workgroup used a revised SCC instrument for the Coatesville SCC self-assessment, striving for plain language. We combined social support and sense of community into “connectedness,” defined as “a sense of belonging, neighborliness, and demonstrated patterns of sharing and caring for others.”

### 3.4. Striving for Comprehensiveness, a Community-Based Self-Assessment Process Must Still be Practical by Acknowledging Participants’ Limited Time and Competing Obligations

Phase 1 document review revealed that the time required by community resilience self-assessment tools could span from several hours to many months and involve either very small or large groups of assessors [39,41,43]. For instance, one toolkit encourages select community leaders to download a 19-page workbook and expeditiously identify weaknesses they want to address prior to the next hazard event [43]. By contrast, another offers a more comprehensive set of data collection and group process tools (e.g., survey questionnaire, key informant interview guides, neighborhood infrastructure maps, capacity and vulnerability assessment), allowing for a broader community engagement process [39,40]. Observations made during Phase 3 pilot testing included the finite time that diverse individuals could invest in the assessment process at one sitting and the steady tempo required to cycle group discussion from a basic orientation to concepts and process, to a collective rating exercise, to a formulation of next steps, to designating priority activities and actors to carry them out.

### 3.5. Who Conducts the Community Resilience Rating and What Evidence or Rationale is Used to Support Their Rating will Affect a Score’s Legitimacy

Scoring needs to be seen as legitimate, i.e., a genuine reflection of the state of the domains being assessed. Phase 1 discussions among the entire COPEWELL team revealed diverse opinions about the objectivity of the self-rating exercise. Some team members advocated that the rubric incorporate sample metrics for users to consider and encourage users to identify pre-existing quantitative data to support their ratings whenever possible, moderating bias. Other team members indicated that the self-assessment’s authority derived more from the collective discussion. Phase 2 stakeholders indicated that it would be important to have a third party facilitate the scoring because grading one’s self objectively is difficult; others indicated that community users should “own” the process themselves. In either case, “who” does the scoring and how people weigh in on the rating will affect its legitimacy. For some people, legitimacy comes from objective data; for others, legitimacy comes from collective engagement with diverse rationale. 

### 3.6. Community Resilience Assessment Tools Must Be Paired with “Promising Practices” and Potential Interventions

Scoring a domain that influences community resilience should not be an activity unto itself, but instead, drive people toward solutions. Phase 1 review of existing self-assessment tools demonstrate that some developers have compiled resources that outline specific interventions to increase resilience. Phase 2 stakeholders strongly encouraged rubric developers to identify a suite of “promising practices” through which communities could enhance their resilience in specific domains. Phase 3 field test participants indicated that ratings should naturally lead to an “improvement plan.” One local participant, for instance, asked, “We already know what the problems are, so what do we do about it?” Preparedness summit attendees also suggested that, when presenting users with potential interventions, rubric developers should also point to potential grants and other funding sources that could support the work.

### 3.7. Not All Community Resilience Domains Are Created Equal (i.e., They Operate at Different Geo-Scales), Prompting the Need for Different Assessment Approaches and Assessors

Comprised of 19 interconnected domains, the COPEWELL model represents a complex reality (Figure 1). All domains, however, do not neatly align in terms of the people, space, structures, and dynamics each incorporates. A single neighborhood or small municipality, as in the case of Coatesville, PA, can legitimately assess its own social capital and cohesion; at the same time, that same smaller community sits in a more expansive community (e.g., Chester County and beyond) that various critical infrastructures serve. A distinct set of assessors and evidence will be necessary to conduct a proper assessment of the critical infrastructure domain.

## 4. Discussion

The Composite of Post-Event Well-being (COPEWELL) is a conceptual and computational model that relies upon publicly available, county-level quantitative data to predict community resistance, recovery, and post-event functioning following disasters, providing a comparative national snapshot [35,36]. It constitutes an objective or top-down approach to community resilience measurement [17,18,19,20,30,31,32,36]. By contrast, the COPEWELL Rubric—the development of which is chronicled here—constitutes a more subjective, bottom-up approach to community resilience assessment, while benefitting from the analytic rigor behind the original COPEWELL conceptual model. Aligning with an “objective characterization and subjective evaluation” form of resilience assessment, potential benefits include greater ease of use, relevance to the local context, and public buy-in; potential risks include the influence of cognitive biases [31]. 

Phases 1–3, as outlined in the Material and Methods section, represent major steps in the larger COPEWELL Rubric development process (Figure 2). One significant outcome achieved thus far is a set of field-tested materials with which communities can advocate a common vision of community resilience as a concrete, desirable, and obtainable goal, and with which they can assess and simultaneously strengthen their social capital and cohesion—an established pathway to withstand and recover from disasters more effectively [11,30,38,39,40,41]. At the outset of the Chester County, PA partnership, the workgroup had begun developing other domain-specific instruments for self-assessment, and then pivoted to the focus on SCC, because that this was the community’s self-identified priority. Once complete, the workgroup intends to test the entire rubric (i.e., the full set of self-assessment instruments) with a new community partner in another state who is interested in applying the complete COPEWELL framework for community resilience. 

Based on the results detailed in the previous section, the rubric workgroup has adopted the following development priorities moving forward.

### 4.1. Anchor the Rubric More Securely within a Larger Visioning-Planning-Acting Cycle

**Figure 4:**
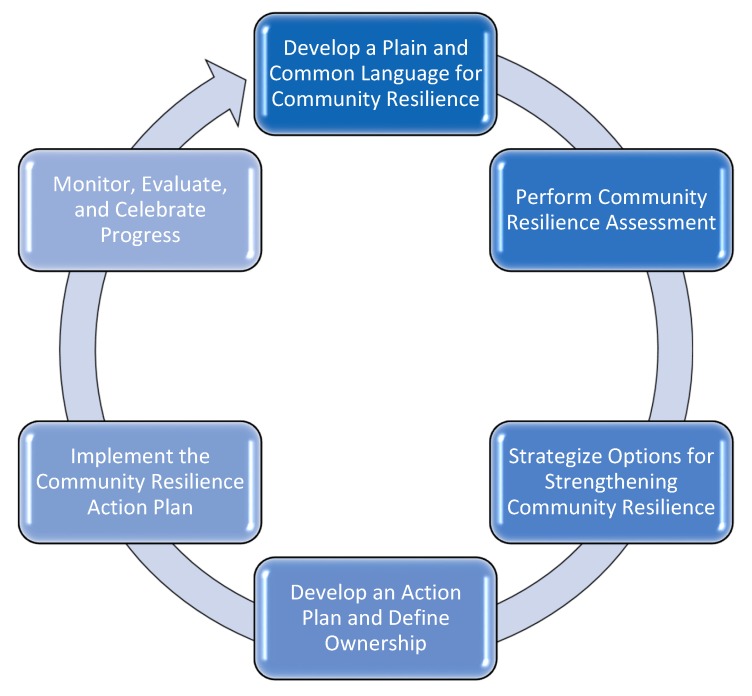
Participatory Visioning-Planning-Acting Cycle * using the COPEWELL Rubric to Enhance Community Resilience (Adapted from References [41,47]).

Key benefits to communities of systematically assessing their resilience include a common frame of reference (or language) to support dialogue about collective well-being before, during, and after a disaster; greater awareness about the strengths and deficits impinging upon the community’s ability to withstand and recover from a disaster; more informed decisions about which gaps to address first and with what resources; and the means to track, communicate, and celebrate success more readily [23,25,26]. Developing the core assessment instruments has been the rubric workgroup’s initial focus, and stakeholders have vigorously indicated that assessment must lead to action: Why measure current status, if not to advance further? Our pilot community’s desire for guidance on post-assessment activities (e.g., which interventions to adopt that would strengthen their resilience) matches the experience of other localities eager to translate assessment findings into concrete decisions and actions that enhance resilience [21]. The rubric workgroup thus plans to develop materials that enable communities to engage in a full cycle of visioning-planning-acting (Figure 4) [41,47]. These include user guides that instruct in good process, connect the assessment work to other community enhancing enterprises, and outline where and how to identify best practices for enhanced resilience. 

### 4.2. “Crosswalk” the Rubric with Extant Grant Guidance and Promising Practices

Comprehensive reviews of the community resilience assessment field reveal that many scholarly-informed tools exist, yet few are actually implemented [17,21,22,23,29]. Among the factors impeding use of existing schemes are their variance with local decision-making, unsuitability for low-resource environments, and disproportionate focus on characterization rather than action [17,21,22,28,33,34]. Recognizing that community resources (e.g., time, money, political will, expert availability, resident interest) are limited, and other priorities compete for attention, the COPEWELL team will work to refine the rubric so that its application conserves and uses local resources wisely. For its next iteration, for instance, the workgroup will—at stakeholders’ recommendation—crosswalk the rubric against current grants (e.g., federal support to state and local health departments for community preparedness), showing users how community resilience assessment relates to current activities and planning cycles. Making the rubric relevant to ongoing obligations can prevent its application from becoming a one-off, stand-alone, and/or resource-intensive enterprise with no discernible impact. Moreover, featuring promising practices in the rubric user guides can better move users to action.

### 4.3. Enhance the Rubric’s Usability and Use

A persistent challenge for community resilience assessment is the balance between conceptual rigor and operational detail [19,23,24]. A 2019 comprehensive review of current resilience measurement efforts (and paucity of their use) noted a “conundrum”: tension between simplistic, user-friendly, yet adversely streamlined methodologies and complex, valid/reliable, yet ungainly ones [21]. In the COPEWELL case, the rubric workgroup had originally intended to develop assessment instruments for all 19 domains in the original model (Figure 1). However, based on community feedback about the need for an efficient, user-friendly tool, we have distilled the approach to six more economical, self-assessment instruments. Now drafted, a new Community Functioning instrument, for instance, collapses ten of the COPEWELL model’s original domains into one with five sub-factors: life necessities, health/well-being, economy, government, critical infrastructure. Jurisdictions that require more granular treatment of the original ten domains will be referred to more sophisticated sector-specific assessment materials, when they exist.

### 4.4. Keep the COPEWELL Approach to Self-Assessment Flexible

A limitation of current approaches to evaluating and improving community resilience is their inflexibility, having been tailored to a specific kind of community [23,24,34], and having assumed a certain resource level (e.g., staff, time, budget) available for their application [21,33,34]. The rubric workgroup is therefore producing user guides to allow a community to apply the rubric on its own, normally with no need for hired assistance. The goal is to produce a tool that jurisdictions of varying resource-levels can implement for their own purposes. The user guides will include one for each of the six rubric instruments as well as an “umbrella” guide that familiarizes a community with basic COPEWELL concepts and self-assessment approaches. The user guides, too, will suggest different meeting time-frames and formats (e.g., two-hour rapid assessment, advance data collection and one-day rating workshop) so that communities can customize the rubric to their situation.

### 4.5. Strengthen the COPEWELL Rubric as a Convening, Coordinating, and Collaborating Tool

A recent review suggests that extant community resilience assessment tools are uneven in their ability to improve the capacity of communities to anticipate, absorb, recover from, and evolve after an extreme event [48]. In this analysis, assessment tools do not just have a strictly instrumental value, say, for gauging the level of a community’s resilience; they also have generative properties. Participatory approaches to community resilience assessment, for instance, can prompt more innovation, self-organization, social learning, and collaborative planning—properties of a community better able to adapt to an adverse event [48]. As a bottom-up, participatory approach to assessment, the COPEWELL Rubric is itself an intervention—not just something that points people to other domain-specific interventions—that can elevate qualities associated with greater community resilience. Therefore, the workgroup is approaching the users guides as an essential element of the toolkit, in that they can facilitate a higher quality process of cross-sector understanding and collaboration. These users guides, for example, will include concrete guidance on the “how and why to do an assessment,” such as who to bring to the table, how to come to a consensus, and how to chart a course forward.

## 5. Conclusions

Due to persistent population vulnerabilities and an increasingly hazardous environment, the desire and need for community resilience to disasters have intensified. Against this setting, the science of community resilience has evolved in emphasis from delineating basic definitions, to developing models and measurement schemes, to disseminating assessment tools to field users. Despite their abundance and variety, however, community resilience measurement schemes are not as popular among users as their developers would hope. Tensions between academic values of reliability and validity and practitioner virtues of utility and relevance explain the implementation deficit, in part. Still, more detailed empirical study of the application of community resilience indicators could shed further light on the academic-practice tension and other obstacles to more widely performed assessments. Moreover, closer and earlier research-practice-community collaborations, too, could help generate more useful and used measures.

Given the need to understand the implementation of community resilience measures better, this article chronicles the co-development of the COPEWELL Rubric with community-based users and national thought leaders. The rubric constitutes a participatory, bottom-up approach to community resilience assessment. Upon implementation, potential community benefits include broader risk and resilience awareness, greater local ownership of the evaluation and action planning, enhanced social capital and a greater capacity to solve problems collectively [11,30,31]. These benefits accrue to a community’s preparedness and ability to mitigate the consequences of disasters, disease outbreaks, and other adverse public health occurrences. At the same time, community feedback has pushed the COPEWELL Rubric workgroup towards needed improvements to the resilience assessment tool, including greater attention to materials (e.g., user guides for process and best practices) that would facilitate ease of application as well as the seamless transition to action. 

## Supplementary Materials

The following are available online at https://www.mdpi.com/1660-4601/16/13/2372/s1, Figure S1: Workshop Agenda for Simulated Self-Assessment Among Public Health Preparedness Experts Using Draft Rubrics for 3 COPEWELL Domains—April 17, 2018; Figure S2: Facilitator and Participant Guide for Simulated Self-Assessment Sessions Held Among Public Health Preparedness Experts—April 17, 2018, Figure S3: Agenda for Self-Assessment Workshop Held Among County-Level Stakeholders Using the Draft Social Capital and Cohesion Rubric—July 27, 2018; Figure S4: Facilitator’s Guide for the Workshop Held Among County-Level Stakeholders for Self-Assessment using the Social Capital and Cohesion Rubric—July 27, 2018; Figure S5: Agenda for Self-Assessment Workshop Held Among City-Level Stakeholders Using the Draft Social Capital and Cohesion Rubric—October 18, 2018; Figure S6: Facilitator’s Guide for the Workshop Held Among City-Level Stakeholders for Self-Assessment using the Social Capital and Cohesion Rubric—October 18, 2018.
