# Supplementary material for: The COPEWELL Rubric: A Self-Assessment Toolkit to Strengthen Community Resilience to Disasters
Source: Int J Environ Res Public Health. 2019 Jul 4;16(13):2372. doi: 10.3390/ijerph16132372 (PMC6651431; doi:10.3390/ijerph16132372)
Supplement: Supplementary file 1 [file ijerph-16-02372-s001.zip › Figure/Figure S3.docx]

**Figure S3.** Agenda for Self-Assessment Workshop Held Among County-Level Stakeholders

Using the Draft Social Capital and Cohesion Rubric – July 27, 2018

**COPEWELL and Resilient! Chester County –**

**Application of the “Social Capital and Cohesion” Rubric**

**Workshop Description**: Key informants from Chester Co will pilot the application of one component of the COPEWELL self-assessment tool -- the Social Capital and Cohesion Rubric. This will be a facilitated community-based discussion aimed at rating the county’s capability and capacity around social capital and cohesion, stimulating ideas about ways to strengthen this domain, and starting to identify priorities and players for doing that work.

**Simulation Objectives**:

- Gain insights into how the Social Capital and Cohesion Rubric can best be applied / used / adapted for use by local Chester Co communities that have varying levels of interest and expertise in resilience;
- Elicit practical feedback on process improvements and supporting materials that the COPEWELL project will need to develop so that communities can ideally apply the rubric on their own.

**Schedule:**

| 9:00-9:05am | **Introductions:** Who’s here? What is “social capital and cohesion” and where does it fit in the COPEWELL model? How is development of the full self-assessment tool proceeding? |
| --- | --- |
| 9:05-9:10am | **Discussion Set Up**: How will we use the rubric? What are the different components? What are discussion ground rules? |
| 9:10–9:55am | **Capturing our Collective Wisdom: Social Capital and Cohesion Self-Assessment**  **Sub-factor #1:** Social support (9:10-9:25am) |
|  | **Sub-factor #2:** Sense of community (9:25-9:40am) |
|  | **Sub-factor #3:** Citizen participation (9:40-9:55am) |
| 9:55-10:10am | **Generating Possibilities**: What ideas for strengthening Social Capital and Cohesion does this conversation spark? Are there activities already underway or being considered that can boost this domain? Which one(s) can and should we advance first? Who cares enough about each activity to own or partner in implementing it? |
| 10:15-10:45am | **Debrief**: What did you like about today’s discussion? What was challenging? Are there things to adapt in the tools or process? What supports or tools would enable you and Chester Co. communities to apply the rubric process yourselves, locally? |
| 10:45-11:00am | **Next Steps in Chester Co:** Which Chester Co communities could get excited about or benefit from applying the rubric? Which could we best learn from? Who wants to / should be involved? Where do we go from here? |
