# Supplementary material for: The COPEWELL Rubric: A Self-Assessment Toolkit to Strengthen Community Resilience to Disasters
Source: Int J Environ Res Public Health. 2019 Jul 4;16(13):2372. doi: 10.3390/ijerph16132372 (PMC6651431; doi:10.3390/ijerph16132372)
Supplement: Supplementary file 1 [file ijerph-16-02372-s001.zip › Figure/Figure S2.docx]

**Figure S2.** Facilitator and Participant Guide for Simulated Self-Assessment

Sessions Held Among Public Health Preparedness Experts – April 17, 2018

**The COPEWELL Rubric: A Self-Assessment Tool to Help Strengthen Community Resilience**

**Small Group Exercise – Participant Guide**

**Exercise Purpose**: Simulate application of a COPEWELL scoring rubric in the form of a community-based discussion to rate a jurisdiction’s capability and capacity in a specific domain (e.g., social capital, natural systems, public health/health care).

**1. Form Groups for Simulated Community Dialogues**

- Gather with others (preferably 7-9 total) to discuss one domain among the 3 possible ones.

**2. Define Your Community**

- Recognize that you come from varied jurisdictions, yet will pretend you are all from one common community. Draw on your own knowledge and experiences to bring authenticity and plausibility to your fictional community.
- Decide on the nature of your fictional locale – frontier, rural, suburban, urban.
- Determine the geoscale and specific “community” you are discussing in relation to your specific domain. You may choose a county, zip code, school district, neighborhood, etc.
- State your reasons for why the geoscale is appropriate for the domain you have chosen.

**3. Determine the Stakeholders at the Table**

- Share your thoughts on which agencies and actors would constitute the best group of stakeholders to assess your chosen domain. During the simulation, consider adding in the kind of input that would be provided by this optimal group of community perspectives.
- Feel free to role play as a knowledgeable community member or a State / Fed official helping the community to self-assess.

**4. Familiarize Yourself with the Scoring Rubric Components** (facilitators will assist)

- Review the domain definition and explanations of the sub-factors that constitute that domain.
- Select one factor around which to direct your fictional discussion.

**5. Engage in a Simulated Dialogue to Self-Assess a Chosen Factor**

- Review the questions listed for that factor and use them to prompt discussion on the status of this factor in your chosen community. You do not need to limit yourself to these questions or to address every question sequentially.
- Talk about:
- Data available: What community data do you have available on this factor, if any, and what does it suggest?
- Community wisdom/experience: What do you believe to be true about this factor from personal or professional experience/observation, participation in the community? What are community strengths in this area? Gaps? Give examples supporting your thoughts?
- Additional info needed and attainable, if any: Who else, if anyone, needs to be at the table and/or provide input for consideration? Identify any other info relevant to understanding this factor in your community.
- Drive toward a consensus rating. Review “low” vs “optimal” capacity descriptions for the factor you discussed. Discuss where you think your chosen community falls on that spectrum.

**Self-Reflection and Evaluation of Rubric Discussions**

**Feedback on Scoring Rubric**

The rubric includes various components (e.g., domain definition, defined sub-factors, self-reflection questions, low-to-optimal capacity descriptions). Do you have any feedback on the structural components themselves in terms of their usefulness to drive understanding, engagement, and assessment data?

- Does breaking down the domain into discrete sub-factors help make the domain more intelligible and easier to discuss and rate as a group? Why or why not?
- Do the prompting questions for each sub-factor encourage a robust self-assessment? Do they help drive in the direction of a consensus rating? Why or why not?
- Do you feel as if the specific scoring rubric you used successfully captured the key factors or elements of that domain? Was anything major missing?
- Does having a low and optimal capacity rating description help you define a continuum upon which to rate your community?
- What else does the prototype tool need in order to be useable and salient in the field (e.g., more graphics, less text, online format)?

**Implementation Process Ideas**

- Based on your experience with other self-assessment activities, are there other elements of self-assessment that need to be built into the COPEWELL scoring tool?
- Can you envision the COPEWELL scoring tool being implemented in your own jurisdiction successfully, why or why not?
- To what degree would the following things have to be in place in order to conduct a self-assessment using COPEWELL scoring rubric(s): process leadership, interagency coordination, community buy in, dedicated staff, data to back up assessment? Anything else?
- Do you think that each of the 3 scoring rubrics (i.e., social capital, natural systems, public health/health care) would require a different set of stakeholders to obtain a credible rating? Why or why not? If different stakeholders indeed were needed to rate the various domains, how could a jurisdiction best maintain a comprehensive big picture of its potential resilience to disaster?
- Are there any routinely done self-assessment activities in your jurisdiction with which the COPEWELL self-assessment could be coordinated for greater efficiency and synergy?
- State or Fed officials: How might a COPEWELL-driven self-assessment fit into or support a broader assessment of resilience as a state or nation?
