# Supplementary material for: The COPEWELL Rubric: A Self-Assessment Toolkit to Strengthen Community Resilience to Disasters
Source: Int J Environ Res Public Health. 2019 Jul 4;16(13):2372. doi: 10.3390/ijerph16132372 (PMC6651431; doi:10.3390/ijerph16132372)
Supplement: Supplementary file 1 [file ijerph-16-02372-s001.zip › Figure/Figure S5.docx]

**Figure S5.** Agenda for Self-Assessment Workshop Held Among City-Level Stakeholders

Using the Draft Social Capital and Cohesion Rubric – October 18, 2018

**What Everyday Neighborliness Means for Disasters:**

A Workshop to Measure and Strengthen How Well Coatesville

Pulls Together as a Community, and as a Result, Bounces Back from Disasters

**Sponsored by COPEWELL and Resilient! Chester County**

**October 18, 2018**

**Description**: Key informants from Coatesville will discuss how everyday neighborliness and community participation (aka “social capital”) can also help a city, town, or county bounce back from a disaster (ie, be “resilient”). Using a tool or “rubric” developed by a mix of grassroots and academic experts, participants will measure how well Coatesville residents pull together as a community and then identify specific ways to strengthen this collective trait. The long-term aim is to help reduce the impacts of future disasters. The workshop is part of an on-going partnership between the COPEWELL project and Resilient!Chester County to develop and test resources that help local communities better withstand disasters, while seeing benefits today.

**Objectives**:

- Equip residents with a structured way to rate or score, as a group, Coatesville’s ability to pull together as a community and to track this trait over time (to see if it is improving)
- Motivate a dialogue about what concrete actions Coatesville can take to strengthen everyday neighborliness and community participation, drawing on current assets and attending to gaps
- Initiate “next steps” planning whereby residents identify who or what entities can take on the work of strengthening social capital as well as where to start first
- Enable the COPEWELL team to discover needed improvements for the rubric and users guide so that other communities can find value in the project’s products for fostering disaster resilience

**Schedule:**

| 2:00-2:10pm | **Introductions:** Who’s here? Why are we here? What is the COPEWELL model of resilience? How do neighborliness and community participation (aka “social capital”) help a town or city bounce back from disasters? |
| --- | --- |
| 2:10-2:15pm | **Discussion Set Up**: What is a rubric? How will we use it? What are the different components? What are discussion ground rules? |
| 2:15–3:10pm | **Capturing our Collective Wisdom:** Social Capital Rating/Rationale  **Individually:** Neighborliness & Community Involvement (2:15-2:20pm)  **Group Assessment**: Neighborliness (2:20-2:45pm)  **Group Assessment**: Community Involvement (2:45-3:10pm) |
| 3:10-3:55pm | **Generating Possibilities**: What ideas for strengthening social capital does this conversation spark? Are there activities already underway or being considered that can boost this domain? Which one(s) can and should we advance first? Who cares enough about each activity to own or partner in implementing it? |
| 3:55-4:00pm | **Thanks and Debrief (survey):** What did you like about today’s discussion? Are there things to adapt in the tools or process? What else could help other communities like Coatesville hold this discussion? |
