# Supplementary material for: The COPEWELL Rubric: A Self-Assessment Toolkit to Strengthen Community Resilience to Disasters
Source: Int J Environ Res Public Health. 2019 Jul 4;16(13):2372. doi: 10.3390/ijerph16132372 (PMC6651431; doi:10.3390/ijerph16132372)
Supplement: Supplementary file 1 [file ijerph-16-02372-s001.zip › Figure/Figure S4.docx]

**Figure S4.** Facilitator’s Guide for the Workshop Held Among County-Level Stakeholders

for Self-Assessment using the Social Capital and Cohesion Rubric – July 27, 2018

**COPEWELL and Resilient! Chester County –**

**Application of the “Social Capital and Cohesion” Rubric**

**Facilitators’ Guide**

**Workshop Description**: Key informants from Chester Co will pilot the application of one component of the COPEWELL self-assessment tool -- the Social Capital and Cohesion Rubric. This will be a facilitated community-based discussion aimed at exploring and eventually coming to consensus around a self-assessed rating of the county’s level of *social capital and cohesion*. The dialogue is also intended to stimulate ideas about ways to strengthen the community’s Social Capital and Cohesion and how priority efforts might be advanced.

**Discussion Objectives**:

- Gain insights into how the Social Capital and Cohesion Rubric can best be applied / used / adapted for use by local Chester Co communities that have varying levels of interest and expertise in resilience;
- Elicit practical feedback on process improvements and supporting materials that the COPEWELL project will need to develop so that communities can ideally apply the rubric on their own.

**Resources:**

- Participant Handouts: Agenda, COPEWELL Diagram; SCC Rubric
- Facilitator Tools: Facilitator Guide
- Slide projector or print outs
- Brief intro slides adapted for your audience
- Drinks and Refreshments if desired.

**Roles (one person may fill multiple roles):**

- Person to Open / Set the Stage
- Discussion Facilitator
- Flip chart note capturer (helps people see their ideas captured / refer back to items)
- Seat Note taker (may use with flip charts if want to capture more detail
- Time keeper / additional discussion prompter
- Evaluator, if applicable.

**Room Set Up:**

- Room should be comfortable for participants and conducive to dialogue / safe sharing with each other. Chairs may be set in a U shape or circle. You’ll need to decide if all participants will take part in a single dialogue, or if you will break into groupings (may need to do with groups of >15-20)

**Presentation and Discussion Guide:**

1. **Introductions and Overview:**

- Who’s here? – do brief intros among the participants (or distribute a list of participants).
- *Go over the intro slides to briefly share what the COPEWELL model is and to introduce what “social capital and cohesion” is / where it fits in the COPEWELL model? With this particular rubric topic, briefly explain that Social Capital and Cohesion is actually a proxy for emergent collective behavior after disasters – the things friends, neighbors, citizens mobilize themselves to do / do for each other during and in the aftermath of a disaster.*

1. **Discussion Set Up:** This is a discussion that a community of any size can hold, however the larger the jurisdiction, the less granular or representative some information may be. Recognize that **for today’s purposes, the discussion will be a bit artificial** -- because you each come from diverse sub-jurisdictions and communities within Chester County. **Ultimately, you’d be holding this discussion with smaller Chester County communities**. (Recognizing that even with smaller jurisdictions, people’s experiences are varied)

Today, you’ll be discussing the **county as a whole**, drawing on your own **knowledge and experiences** both **professionally and as a citizen** living here

*Review the* ***Rubric structure and walk folks*** *through the flow of what they’ll be looking at. Explain the parts and the flow of how they will use it. (Definition, Subcomponents, Questions, Low/Optimal Capacity Descriptions, and Rating scale / Rationale Capturing.)*

So in sum, we are looking to hold a **discussion on social capital and cohesion in Chester Co** -- to **describe our community and the experience of its citizens**. From this, we’ll collectively provide an **assessment** of where we stand -- a general concept of how we feel our community is performing in this domain. And finally, we’ll identify **possibilities for strengthening** this aspect of our community.

**Ground Rules for Discussion:**

- **Everyone has something to contribute**. Actively **share** your knowledge, perceptions, experiences. Also work to **draw out** those of others in the group—ask questions, listen closely to understand, etc.
- Not everyone has the same **experience or perception**. All are **valuable and grounded in truth**. This is a dialogue, not a debate.
- There is likely some **quantitative / fact based / example-based information** that can inform the discussion – feel free to reference it, share it, or recommend looking at it. There’s also **perception and experience that is equally valuable**.
- **New York Times, rule**… To give all a chance to be heard, speak in headlines, where possible;

1. **Capturing our Collective Wisdom: Social Capital and Cohesion Dialogue and Self-Assessment**

**(45 min.; 15 min / subfactor)**

- Review the domain definition and explanations of the sub-factors that contribute to the domain. Pick a sub-factor to start with. Review the definition of that factor.
- (On Rubric Back) Review the questions listed for that factor and use them to prompt discussion on the status of this factor in your community. You do not need to limit yourself to these questions or to address every question sequentially. Spend about 15 minutes on the subfactor as a whole.
- *Prompt Discussion about:*
- Data available: What community data are available on this factor, if any, and what does it suggest?
- Community wisdom/experience: What do you believe to be true about this factor from personal or professional experience/observation, participation in the community? What are community strengths in this area? Gaps? Give examples supporting your thoughts?
- Additional info needed and attainable, if any: Who else, if anyone, needs to be at the table and/or provide input for consideration? Identify any other info relevant to understanding this factor in your community.
- (Back on front of the Rubric) Review the “Low” vs “Optimal” capacity description for the factor you discussed. Discuss where you think your community falls on that spectrum. (5 minutes)
- *Repeat this for the other two subcomponents.*

1. **Generating possibilities (15 min)**

What **ideas for strengthening Social Capital and Cohesion** does this conversation spark? Are there **things already in the works / under consideration** this sheds new light on? Which one(s) can and should we **advance first**? **Who cares enough about each to own or partner in implementing it**? *(If it would help your group, reference the model and ask)* What would fill the tank more? What would help the valve open faster / flow at the right rate in disasters? What would build resilience? *Capture the ideas generated and priorities on paper / notes.*

1. **Debrief: What did we learn (30 min). Prompt discussion around:**

- What did you like about today’s discussion?
- What was challenging?
- Are there things to adapt in the tools or process?
- What supports or tools would enable you and Chester Co. communities to apply the rubric process yourselves, locally?
- What types of individuals and community organizations should participate in SCC Rubric discussions? Can you see it being helpful? (with communities at novice, intermediate, or advanced level in thinking about resilience)
- Do there need to be any adaptations of the approach? If so, what would they be?
- Do there need to be any adaptations of the tool supporting those discussions?
- How would you lay the groundwork for this type of community meeting? What types of individuals / organizations would be key to include or invite?
- What would you want outcomes to be from the discussion?

1. **Next Steps in Chester Co: (20 min)**

- Which Chester Co communities could get excited about or benefit from applying the rubric?
- Which could we best learn from?
- Who wants to / should be involved?
- Where do we go from here?

**Thank you to participants, facility, staff!**
