# Supplementary material for: The COPEWELL Rubric: A Self-Assessment Toolkit to Strengthen Community Resilience to Disasters
Source: Int J Environ Res Public Health. 2019 Jul 4;16(13):2372. doi: 10.3390/ijerph16132372 (PMC6651431; doi:10.3390/ijerph16132372)
Supplement: Supplementary file 1 [file ijerph-16-02372-s001.zip › Figure/Figure S6.docx]

**Figure S6.** Facilitator’s Guide for the Workshop Held Among City-Level Stakeholders

for Self-Assessment using the Social Capital and Cohesion Rubric – October 18, 2018

**COPEWELL and Resilient! Chester County –**

**Application of the “Social Capital and Cohesion”**

**(aka Neighborliness and Community Involvement) Rubric**

**Facilitators’ Guide**

**Workshop Objectives**: Organizational and community representatives will apply one component of the COPEWELL self-assessment tool -- the Social Capital and Cohesion Rubric. This will be a facilitated community-based discussion aiming to:

- develop a self-assessed rating of the community’s level of *neighborliness and community involvement, (*serving as a pre-event proxy for emergent collective behavior during and following disasters).
- help participants uncover the connection between everyday community development and disaster mitigation efforts,
- identify one or more priority activities to build upon or initiate as a means of strengthening community resilience,
- Build community connections through shared exploration of a common theme relevant to all,
- Gain insights into how the Social Capital and Cohesion Rubric can best be applied / used / adapted for use by local communities with varying levels of interest and expertise in resilience;

**Resources:**

- Participant Handouts: Agenda, COPEWELL Diagram; SCC Rubric
- Facilitator Tools: Facilitator Guide
- Brief intro slides adapted for your audience
- Slide projector or print outs
- Flip charts
- A computer or notepad on which to take more detailed notes / capture key points electronically
- Automated voting system or sticky notes, pencils and pens, scratch paper.
- Drinks and Refreshments if desired.

**Staffing Roles (one person may fill multiple roles):**

- Person to Open / Set the Stage
- Discussion Facilitator
- Flip chart note capturer (help people see their ideas captured / refer back to items)
- Seat Note taker (use to capture more detail and capture info electronically)
- Time keeper / additional discussion prompter
- Evaluator, if applicable.

**Room Set Up:**

- Room should be comfortable for participants and conducive to dialogue / safe sharing with each other. Chairs may be set in a U shape or circle. You’ll need to decide if all participants will take part in a single dialogue, or if you will break into groupings (may need to do with groups of >15-20)

**Presentation and Discussion Guide:**

1. **Introductions and Overview:** (10 min)

- Who’s here? – do brief intros among the participants (or distribute a list of participants).
- *Go over the intro slides to briefly share what the COPEWELL model is and to introduce what “social capital and cohesion” is / where it fits in the COPEWELL model? With this particular rubric topic, briefly explain that Social Capital and Cohesion is actually a proxy for emergent collective behavior after disasters – the things friends, neighbors, citizens mobilize themselves to do / do for each other during and in the aftermath of a disaster.*

1. **Discussion Set Up**: (10 min) This is a discussion a community of any size can hold, however the larger the jurisdiction, the more variability there may be across subjurisdictions or even neighborhoods. Even in smaller communities, there is often wide variation in actual and perceived experiences, each of which is valid and welcomed.

Today, you’ll be discussing **[jurisdiction] as a whole**, considering the **likely unique nature of its many parts** and the **varied experiences of different populations** living here**. We ask you to** draw on your own **knowledge and experiences** both **professionally** and/or as a **resident of the area.** Where you feel there is qualitative or quantitative **data to support a topic**, please do bring it into the discussion. However, your collective living and working experience is equally if not more important to formal data in this process.

*Review the* ***Rubric structure and walk folks*** *through the flow of what they’ll be looking at. Explain the parts and the flow of how they will use it. (Definition, Subcomponents, Questions, Low/Optimal Capacity Descriptions, and Rating scale / Rationale Capturing.)*

In sum, we are looking to hold a **discussion to more fully understand the neighborliness of those who live and work here with each other and people’s level of involvement in the community**. From that discussion, we’ll collectively develop an assessment of **where we stand** -- a general concept of how we feel our community is performing in this domain -- and identify **possibilities for strengthening** this aspect of our community.

**Ground Rules for Discussion** (if already covered in slide presentation, just offer reminders):

- **Everyone has something to contribute**. Actively **share** your knowledge, perceptions, experiences. Also work to **draw out** those of others in the group—ask questions, listen closely to understand, etc.
- Not everyone has the same **experience or perception**. All are **valuable and grounded in truth**. This is a dialogue, not a debate. Both sharing from your experience and listening to that of others with a beginner’s mind is important.
- There is likely some **quantitative / fact based / example-based information** that can inform the discussion – feel free to reference it, share it, or recommend looking at it. There’s also **perception and experience that is equally valuable**.
- **Step Up / Step Back**…People process and share info differently. If you are one to quickly jump in to share an opinion or idea, do so, but then step back to really listen to others. If you are typically one to think deeply before speaking, jot down a few notes, think about it, but then step up, even if it’s a bit out of your comfort zone, to share. Everyone’s thoughts and experiences are equally important to put on the table.

1. **Individual Assessment:** (5 min)

We are first going to take a quick poll on where you think the community falls on these factors individually and get you to start thinking about why. Don’t worry about being super precise. There will be opportunity to refine and/or change your response subsequently with more information.

- *Review the domain definition and description of the first sub-factor with the group. Ask individuals to read the Low and Optimal capacity description and rate where they see the [jurisdiction] falling. Have people jot down on a piece of paper a few statements about why they chose the rating they did.*
- *Either use an automated voting system or have folks hand in on a sticky note where they would rate the community on the factor.*
- *Do the same for the subsequent factors.*
- *Have someone calculate the factor mean (average), the range (lowest and highest) and the median (middle value when you put all results in order low to high) of the findings while the discussion continues below.*

1. **Capturing our Collective Wisdom: Social Capital and Cohesion Dialogue and Re-Assessment**
2. **min.; 25 min / factor)**
3. **Dialogue (~20 min):**

- Now we’re going to look at this issue collectively.
- (On Rubric Back) *Review the definition of that factor with the group.*
- *Use the questions / question clusters listed for the factor to prompt discussion about the given community. You do not need to limit yourself to these questions or to address every question sequentially. Spend about 20 minutes on the factor as a whole.*

*Prompt Discussion about:*

- Community wisdom/experience: What do you believe to be true about this aspect of the community from personal or professional experience/observation, participation in the community? What are community strengths in this area? Gaps? Give examples supporting your thoughts
- Data available: What community data are available on this issue, if any, and what does it suggest?

1. **Re-Assessment of Factor (~5 minutes)**

- (Back on front of the Rubric) *Reviewing the “Low” vs “Optimal” capacity description for the factor again, and given the information they have at present, have the group reassess where they think the community falls as a whole on this factor (automated voting or sticky notes).*
- *Have the group briefly summarize the key points / rationales that stood out to them from the discussion leading to the rating. What were the “aha’s” or “key findings” that lead them to select the rating they did.*

1. *Repeat this for the other factor(s).*
2. Additional info needed and attainable, if any: How representative do you think your assessment is today? Who else, if anyone, needs to be at the table and/or provide input for consideration? How you might garner their input, if needed.
3. **Generating possibilities (45 min)**

Now, let’s explore what **ideas for strengthening Social Capital and Cohesion** **(Neighborliness and Community Involvement)** this conversation sparks?

1. **Thinking forward 3 – 5 years, what would your community look like** if it was well on its way to **having optimal capacity** in this arena? (5-10 min)
2. Are there **existing efforts you or your organization have underway / under consideration** that could move you in this direction? How could they be **strengthened or leveraged** to **build even stronger relationships among people / groups** or **to catalyze even greater community involvement**?
3. Given what you’ve learned from and about each other, are there **new connections, partnerships, or ideas** this conversation is sparking? What’s possible?

*(If it would help your group, reference the model and ask)* What would fill the tank more? What would help the valve open faster / flow at the right rate in disasters? What would build resilience?)

*Capture the ideas generated and priorities on flip chart paper / notes.*

1. **Which of these captures your attention as a first step? Identify one or more ideas you feel worth building on / exploring further either individually or collectively**. (15 min)
2. **Who or what groups might own or partner in implementing it either for its everyday benefit or for its benefit in building community disaster resiliency?**
3. What are specific **next steps**? Who will do what? *If time:*
   1. What **support**, if any, is needed?
   2. How will **you track advancements made**? **work through challenges** that arise? **Share and celebrate progress**?
4. **Wrap up and Evaluation: (5 min).**
5. Thank participants, facility, and staff for their participation and on-going commitment to the community.
6. Encourage them, before leaving, to connect with someone they’d like to learn more from or share an idea with.
7. Ask all to complete an evaluation

- What did you like about today’s discussion?
- Are there things to adapt in the tools or process?
- What actions, if any, do you or your organization currently take or commit to moving forward that could advance resilience? Are you interested in further work on this?
- What else would help your or other communities organize their thinking around resilience and generate ideas on ways to strengthen it?
