# Supplementary material for: The COPEWELL Rubric: A Self-Assessment Toolkit to Strengthen Community Resilience to Disasters
Source: Int J Environ Res Public Health. 2019 Jul 4;16(13):2372. doi: 10.3390/ijerph16132372 (PMC6651431; doi:10.3390/ijerph16132372)
Supplement: Supplementary file 1 [file ijerph-16-02372-s001.zip › Figure/Figure S1.docx]

**Figure S1.** Workshop Agenda for Simulated Self-Assessment Among

Public Health Preparedness Experts Using Draft Rubrics for 3 COPEWELL Domains – April 17, 2018

**The COPEWELL Rubric: A Self-Assessment Tool to Help Strengthen Community Resilience**

Public Health Preparedness Summit Session Agenda

**Session Description**: Currently in development, the COPEWELL rubric is a new cross-sector self-assessment tool intended to help communities strengthen their resilience through a common vision, a valid measurement system, and a set of practical interventions. Session participants will help test the rubric to ensure it meets practitioner requirements for utility and relevance.

**Learning Objectives**: At the conclusion of this session, attendees will be able to:

1. Demonstrate a new way of thinking about resistance, recovery, and resilience, where the community’s ability to maintain functioning (delivery of goods and services) during and following a disaster event is the principal object of concern.
2. Outline domains and subdomains that influence resilience, as well as identify measures that provide reasonable representation of capabilities or capacity in given domains.
3. Lead others in their jurisdiction in a process of self-assessment (e.g., domain definition, data collection, self-reflection, capacity rating, rationale generation) involving select domains such as social cohesion, natural systems, and health care and public health.

| 10:30am – 10:35am | Introductions – Purpose and Participants |
| --- | --- |
| 10:35am – 11:10am | Briefing on COPEWELL Model with Q&A – Participants will become acquainted with the COPEWELL conceptual framework, domains, and intended uses. Include overview of Rubric Tool – format, proposed process of data collection / use. |
| 11:10am – 11:15am | Set up of exercise – Exercise guide provided |
| 11:15am – 11:35am | Simulated Application of COPEWELL Rubrics – Participants will divide into smaller groups to engage in a simulated community-based discussion exercise. Attendees will use their knowledge and background to rate their jurisdiction’s capability and capacity in one of the COPEWELL domains (e.g., social capital, natural systems, public health) according to a specific scoring rubric. COPEWELL team members will help facilitate conversation. |
| 11:35am – 11:45am | Rubric and Implementation Ideas / Evaluation – Participants will turn from using a specific rubric tool to critiquing it in terms of usability and basic design (e.g., do prompting questions generate dialogue that help an actual domain rating? How could the tool be improved?) and to offering suggestions around effective field implementation processes. |
| 11:45am – 11:55am | Plenary Session – Discuss key takeaways from each small group |
| 11:55 am – 12:00 pm | Session Evaluation and Wrap up |
